# Fosmidomycin, an inhibitor of isoprenoid synthesis, induces persistence in *Chlamydia* by inhibiting peptidoglycan assembly

**DOI:** 10.1371/journal.ppat.1008078

**Published:** 2019-10-17

**Authors:** Jessica A. Slade, Mary Brockett, Raghuveer Singh, George W. Liechti, Anthony T. Maurelli

**Affiliations:** 1 Emerging Pathogens Institute and Department of Environmental and Global Health, College of Public Health and Health Professions, University of Florida, Gainesville, Florida, United States of America; 2 Department of Microbiology and Immunology, F. Edward Hébert School of Medicine, Uniformed Services University, Bethesda, Maryland, United States of America; UC Irvine, UNITED STATES

## Abstract

The antibiotic, fosmidomycin (FSM) targets the methylerythritol phosphate (MEP) pathway of isoprenoid synthesis by inhibiting the essential enzyme, 1-deoxy-D-xylulose 5-phosphate reductoisomerase (Dxr) and is lethal to intracellular parasites and bacteria. The obligate intracellular bacterial pathogen, *Chlamydia trachomatis*, alternates between two developmental forms: the extracellular, infectious elementary body (EB), and the intracellular, replicative form called the reticulate body (RB). Several stressful growth conditions including iron deprivation halt chlamydial cell division and cause development of a morphologically enlarged, but viable form termed an aberrant body (AB). This phenotype constitutes the chlamydial developmental state known as persistence. This state is reversible as removal of the stressor allows the chlamydiae to re-enter and complete the normal developmental cycle. Bioinformatic analysis indicates that *C*. *trachomatis* encodes a homolog of Dxr, but its function and the requirement for isoprenoid synthesis in chlamydial development is not fully understood. We hypothesized that chlamydial Dxr (Dxr_CT_) is functional and that the methylerythritol phosphate (MEP) pathway is required for normal chlamydial development. Thus, FSM exposure should be lethal to *C*. *trachomatis*. Overexpression of chlamydial Dxr (Dxr_CT_) in *Escherichia coli* under FSM exposure and in a conditionally lethal *dxr* mutant demonstrated that Dxr_CT_ functions similarly to *E*. *coli* Dxr. When *Chlamydia*-infected cultures were exposed to FSM, EB production was significantly reduced. However, titer recovery assays, electron microscopy, and peptidoglycan labeling revealed that FSM inhibition of isoprenoid synthesis is not lethal to *C*. *trachomatis*, but instead induces persistence. Bactoprenol is a critical isoprenoid required for peptidoglycan precursor assembly. We therefore conclude that FSM induces persistence in *Chlamydia* by preventing bactoprenol production necessary for peptidoglycan precursor assembly and subsequent cell division.

## Author summary

Dxr is the second enzyme in the methylerythritol phosphate (MEP) pathway of isoprenoid synthesis and is inhibited by the antibiotic fosmidomycin (FSM). We used FSM as a tool to demonstrate that chlamydial Dxr functions similarly to *E*. *coli* Dxr. Furthermore, we demonstrate that FSM is not lethal to *Chlamydia* as in other bacteria and parasites, but instead induces persistence. Several conditions are known to cause *Chlamydia* to leave normal development and enter the persistent state including β-lactam antibiotic exposure, interferon-y exposure, iron limitation and viral co-infection. Our study identifies FSM as a novel persistence inducer and isoprenoid synthesis inhibition as the underlying mechanism of FSM-induced persistence induction. By inhibiting isoprenoid synthesis, we identify isoprenoids, most likely bactoprenol, as critical molecules required for cell division in *Chlamydia*.

## Introduction

*Chlamydia trachomatis* is a Gram negative, obligate intracellular bacterial pathogen responsible for more than 130 million new genital tract infections each year worldwide and is the leading bacterial sexually transmitted pathogen in the United States [1–3]. Consequences of chlamydial genital tract infections include acute symptoms such as urethritis in men and cervicitis in women [4]. Because 50–90% of chlamydial infections are asymptomatic in men and women, many infections are left undiagnosed and untreated [5, 6]. Asymptomatic genital tract infections can become chronic, resulting in prolonged inflammation and subsequent scarring of the genital tissue. Genital tract scarring can lead to complications including infertility and ectopic pregnancy [7].

Chlamydiae engage in a unique biphasic developmental cycle. Chlamydiae enter the host as the infectious, extracellular form called an elementary body (EB). Once an EB enters the host cell, it begins to differentiate into the noninfectious, replicative form called a reticulate body (RB). After several rounds of division, RBs differentiate back into EBs and exit the host cell to infect adjacent cells [8]. The chlamydiae complete the developmental cycle within a membrane bound vacuole known as the chlamydial inclusion. The inclusion provides a protective environment for the dividing, osmotically fragile RBs and an interface for acquiring essential nutrients from the host [9, 10]. In tissue culture, normal chlamydial development can be disrupted by exposure to β-lactam antibiotics, IFN-y -induced amino acid depletion, iron deprivation, and co-infection with Herpes Simplex Virus [11]. These conditions cause RBs to exit normal development and enter a state known as persistence. During persistence, chlamydiae are viable but cease dividing, resulting in enlarged, morphologically distinct RBs characterized as aberrant bodies (ABs). Because persistence halts cell division and subsequently prevents RB to EB conversion, this state is also characterized by a reduction in the production of infectious progeny. Importantly, aberrant morphology and the reduction in EB production are reversible upon removal of the persistence-inducing agent [12, 13].

Persistence is not only a tissue culture phenomenon but has been established in the *Chlamydia muridarum* murine intravaginal infection model [14]. Furthermore, persistent chlamydiae have been isolated from human cervical tissue [15]. The role of persistence in maintaining *Chlamydia* genital tract infection and its contribution to the associated adverse outcomes requires further characterization. However, it seems likely that persistence would allow chlamydiae to withstand periods of unfavorable growth conditions in humans. Understanding the molecular basis for persistence induction is the starting point for determining how persistence might contribute to infection in humans and whether controlling persistence would improve infection outcomes.

**Fig 1:**
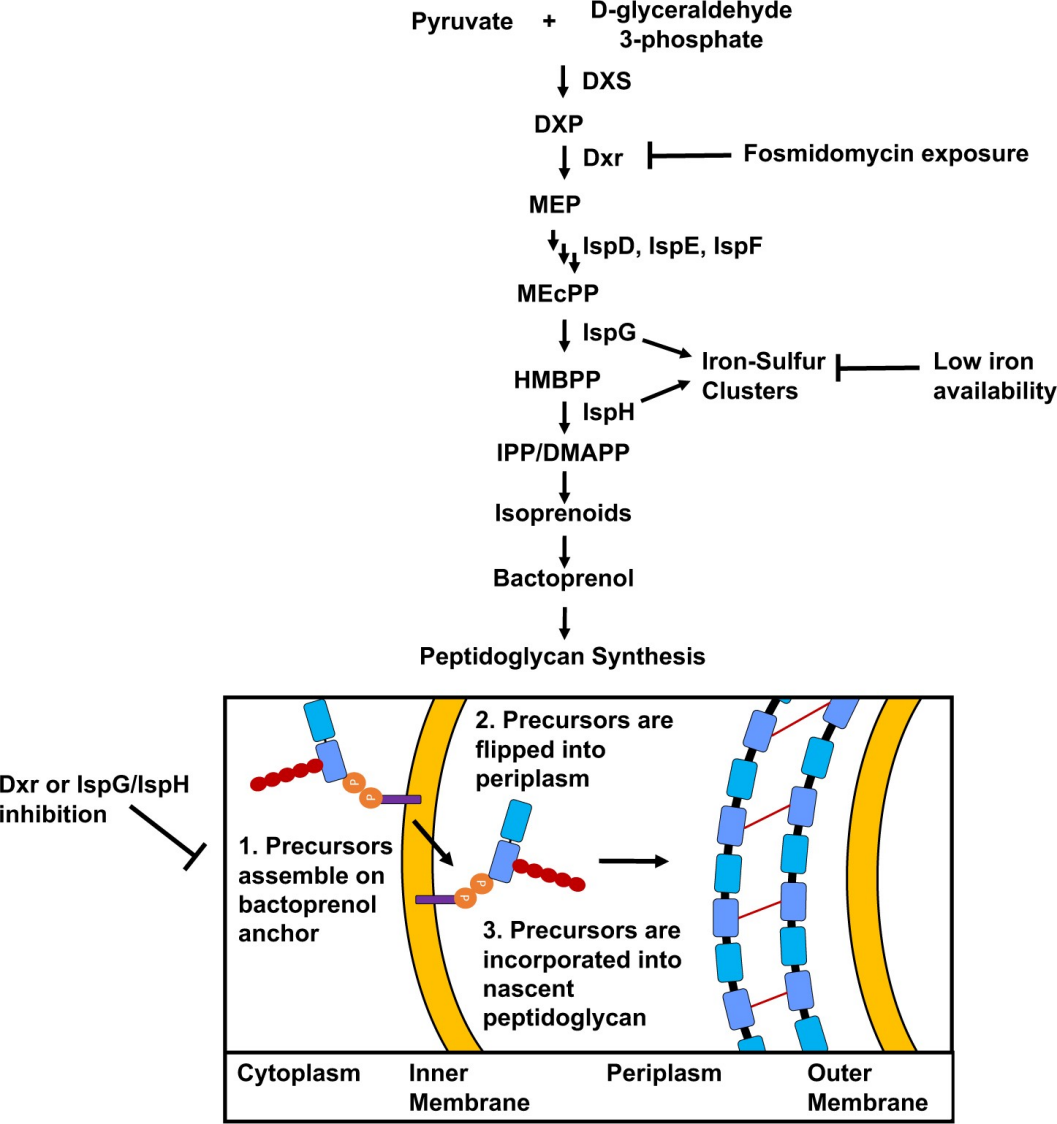
The MEP pathway of isoprenoid synthesis and how its inhibition induces persistence in *C*. *trachomatis*. 1-deoxy-D-xylulose 5-phosphate synthase (DXS) converts initial MEP pathway substrates pyruvate and glyceraldehyde 3-phosphate to 1-deoxy-D-xylulose 5-phosphate (DXP). Dxr converts DXP to MEP. Two additional steps occur generating 2-C-methyl-D-erythritol-2,4-cyclodiphosphate (MEcPP). MEcPP is converted to 4-hydroxy-3-methylbutenyl 1-diphosphate (HMBPP) by IspG. IspH converts HMBPP to the isoprenoid precursor molecules IPP/DMAPP. IPP/DMAPP are stereoisomers produced in a 5:1 ratio and are used to generate isoprenoids. In bacteria, the isoprenoid bactoprenol (purple rectangle), is critical for peptidoglycan synthesis. **1,** Peptidoglycan precursor Lipid II is synthesized in the bacterial cytoplasm through a series of enzymatic reactions and is anchored to the inner leaflet of the inner membrane of the Gram negative bacterial cell envelope via bactoprenol; **2,** The lipid II molecule is flipped into the periplasm; **3,** Periplasmic lipid II is incorporated into the peptidoglycan ring and bactoprenol is recycled. We postulate that both FSM and iron deprivation can disrupt isoprenoid synthesis since IspG and IspH are iron-sulfur cluster-containing enzymes. In both cases, isoprenoid synthesis is halted, bactoprenol availability is reduced and peptidoglycan synthesis is prevented, resulting in persistence induction in *Chlamydia*.

Isoprenoids comprise a vast family of more than 80,000 functionally diverse compounds [16]. The basic structure of an isoprenoid consists of a 5-carbon subunit that can be modified to incorporate additional polycarbon chains. Most isoprenoids are produced by plants, and include compounds such as chlorophyll, carotenoids and vitamins. Substantially fewer varieties of isoprenoids are synthesized by animals and bacteria, but serve critical functions nonetheless. Examples include ubiquinone and dolichols, which are required for energy production via the electron transport chain, and for the generation of glycosylated proteins, respectively. There are two known pathways for isoprenoid synthesis. The mevalonate (MVA) pathway is widely used by all forms of life, whereas, the non-mevalonate pathway, also known as the methylerythritol phosphate (MEP) pathway (**Fig 1**), is used mostly by bacteria, lower eukaryotes, green algae, and some plants [17]. While both of these pathways ultimately produce the isoprenoid precursors isopentenyl pyrophosphate (IPP) and dimethylallyl pyrophosphate (DMAPP), each pathway utilizes a unique series of enzymes and initial substrates. Some bacterial species possess both isoprenoid synthesis pathways, but many, like *E*. *coli* and *C*. *trachomatis*, encode enzymes for only the MEP pathway [18].

**Fig 2:**
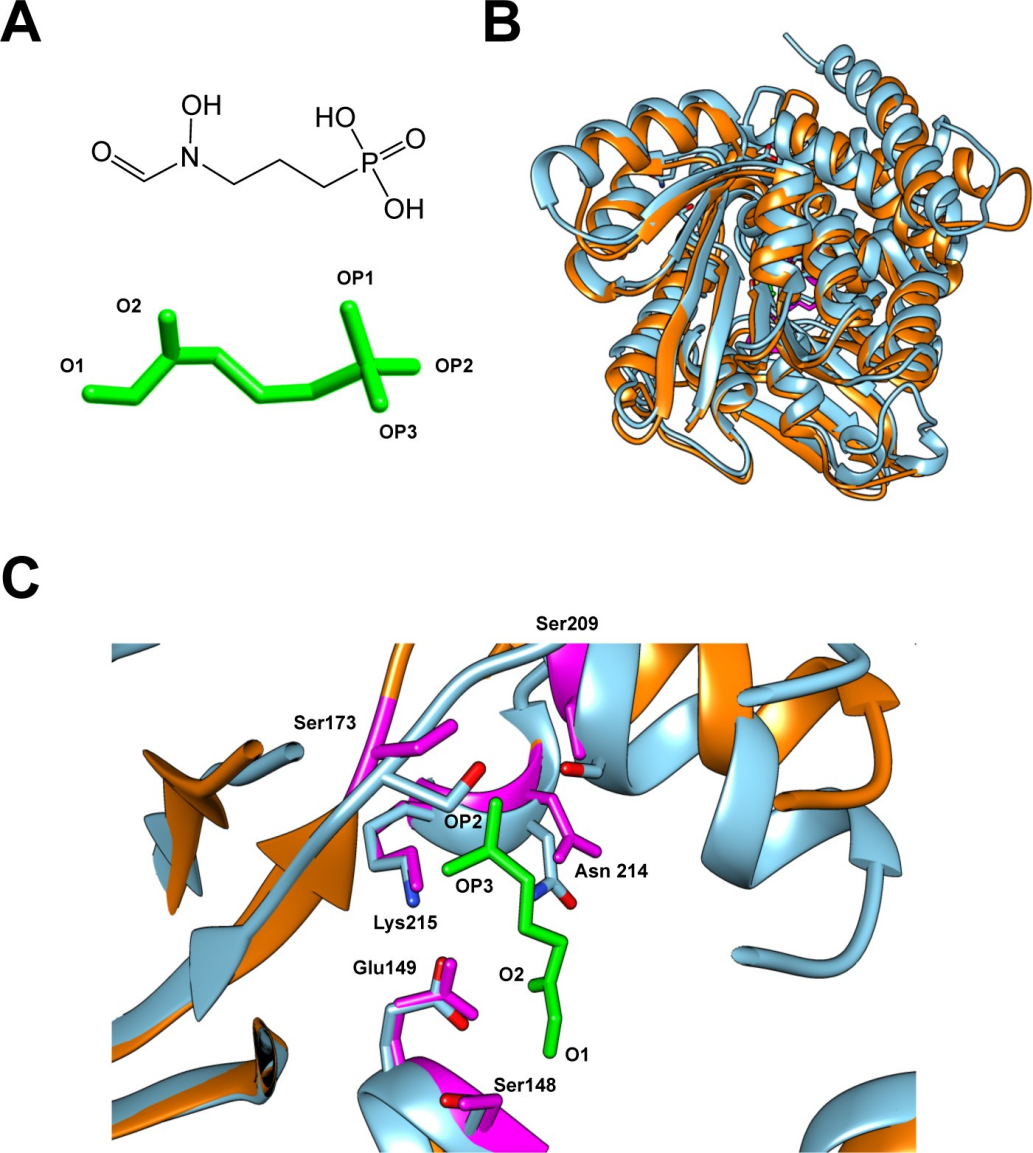
Comparison of the predicted structure of Dxr_CT_ and the crystal structure of Dxr_EC_. (A) Chemical structure (upper) and 3D structure (lower) of fosmidomycin (FSM). 3D FSM structure is in similar orientation to chemical structure, but with reactive oxygen groups labeled (“O” = oxygen; “OP” = oxygen-phosphate). (B) The superimposed view of the predicted crystal structure of Dxr_CT_ (orange) over the crystal structure of Dxr_EC_ (blue). (C) Enlarged view of the FSM binding pocket from the superimposed structures in panel B. The amino acid residues in the binding pocket of Dxr_EC_ (blue) that bind to FSM are conserved in Dxr_CT_ (labeled and highlighted in magenta). FSM is shown in green with OP2, OP3, O2, and O1 sites labeled to indicate orientation.

The MEP pathway converts the initial substrates pyruvate and glyceraldehyde 3-phosphate to IPP/DMAPP through a series of seven enzymatic reactions. Since this pathway is not found in higher eukaryotes, MEP pathway enzymes are enticing targets for treatment of bacterial and parasitic diseases. Fosmidomycin (FSM) is a phosphonic acid antibiotic (**Fig 2A**) produced by *Streptomyces lavendulae* and specifically targets the MEP pathway by acting as a competitive inhibitor of 1-deoxy-D-xylulose 5-phosphate reductoisomerase (Dxr) [19–21]. Dxr, the second enzyme in the MEP pathway, forms homodimers and requires NADPH as a cofactor [20]. Binding of NADPH changes the conformation of the Dxr binding pocket, which promotes both substrate binding and FSM binding [20]. Recently FSM has been used in clinical trials for the treatment of infections with the intracellular parasite, *Plasmodium falciparum*, the causative agent of malaria [22]. However, several bacteria are sensitive to FSM, including multidrug resistant strains of *E*. *coli*, *Enterobacter cloacae*, *Klebsiella pneumoniae*, and *Pseudomonas aeruginosa* [23]. FSM can also inhibit the intracellular growth of bacteria including *Francisella novicida* [19].

Because FSM inhibits the essential enzyme, Dxr and is lethal to bacteria [19, 23, 24], we hypothesized that inhibition of the MEP pathway would be similarly lethal to *C*. *trachomatis*. A previous study reconstructed a partial chlamydial MEP pathway *in vitro* using enzymes downstream of Dxr (IspD, IspE and IspF) to generate MEP pathway intermediates, indicating that the MEP pathway is functional in *Chlamydia* [25]. Here, we used *E*. *coli* as a surrogate system and FSM as a tool to demonstrate that *C*. *trachomatis* encodes a functional Dxr. However, we found that FSM exposure is not lethal to *Chlamydia*, but instead induces persistence in HeLa cell cultures. We postulate that the depletion of isoprenoid precursors leads to shortage of the critical isoprenoid, bactoprenol. Bactoprenol is required for peptidoglycan precursor assembly and ultimately, cell division. Therefore, we conclude that FSM-induced isoprenoid synthesis inhibition leads to a shortage of bactoprenol that ultimately prevents cell division and causes persistence induction.

## Results

### Modeling of chlamydial Dxr predicts a structure similar to *E*. *coli* Dxr

Due to the lack of a crystal structure for *C*. *trachomatis* Dxr (Dxr_CT_), homology modeling was used to generate a predicted structure. The web-based portal Phyre2 generated the structure of Dxr_CT_ with 100% modeling confidence, suggesting that the modeled sequence is likely to adopt the 3D structure and could therefore predict the binding pocket for FSM. To investigate the binding site for FSM, the predicted Dxr_CT_ structure was superimposed on *E*. *coli* Dxr (Dxr_EC_) crystalized with FSM (**Fig 2B**). Based on the 3D structural alignment, Dxr_CT_ demonstrates 37% identity to Dxr_EC_ (**S1 Fig**). However, the most critical region, the putative FSM binding pocket, aligned well with the FSM binding pocket of Dxr_EC_ (**Fig 2C**). The *E*. *coli* residues Ser 151, Ser 186, Ser 222, Lys 228, Asn 227, and Glu 152 form critical hydrogen bonds with either the oxygen (O) or oxygen-phosphate (OP) groups of FSM [20]. FSM OP1 binds with Lys 228 and Asn 227; FSM OP2 binds to Ser 222 and Ser 186; and FSM OP3 binds to Lys 228. FSM O1 binds to Ser 151; and FSM O2 binds with Glu 152 and Asn 227. Importantly, all of the critical residues in Dxr_EC_ that make bonds with FSM were present in the binding pocket of Dxr_CT_. These residues correspond to Ser 148, Ser 173, Ser 209, Lys 215, Asn 214, and Glu 149 in the predicted Dxr_CT_ crystal structure, suggesting that Dxr_CT_ is capable of binding to FSM.

### Expression of chlamydial *dxr* supports growth of a conditionally lethal *E*. *coli dxr* mutant

**Fig 3:**
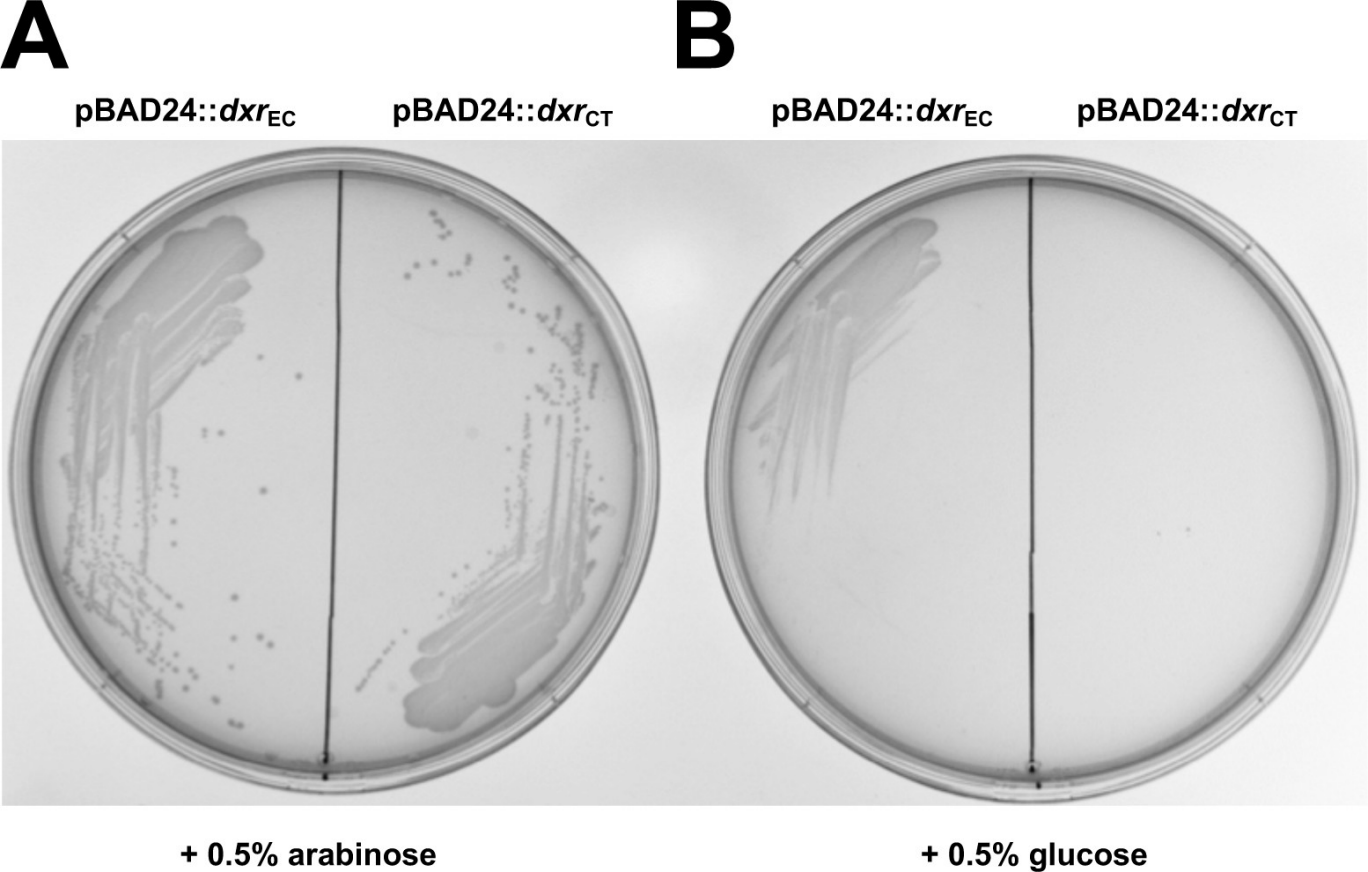
Expression of Dxr_CT_ *in trans* supports growth of an *E*. *coli dxr* conditionally lethal mutant. Five μL of an overnight culture of *E*. *coli* MG1655 Δ*dxr*::*kan* pBAD24::*dxr*_EC_ (ATM1471) or *E*. *coli* MG1655 Δ*dxr*::*kan* pBAD24::*dxr*_CT_ (ATM1470) grown in the presence of 0.2% arabinose were streaked for isolation on an LB agar plate containing 0.5% arabinose (A) or 0.5% glucose (B). Images are representative of three independent experiments performed in duplicate.

Deletion of *dxr* renders bacteria auxotrophic for the intermediate metabolite, 2-*C*-methyl-D-erythritol (ME) [26, 27]. Due to the high cost and limited availability of ME, we began characterizing the function of Dxr_CT_ by employing conditional lethal *dxr* mutants of *E*. *coli*. When induced by the addition of 0.5% arabinose, *E*. *coli* MG1655 Δ*dxr*::*kan* strains transformed with pBAD24::*dxr*_CT_ (ATM1470) and pBAD24::*dxr*_EC_ (ATM1471) (**Table 1**) formed colonies on solid media (**Fig 3A**). Upon suppression of expression of either Dxr_EC_ or Dxr_CT_ using 0.5% glucose, a loss of colony formation was observed (**Fig 3B**). Since *E*. *coli* MG1655 Δ*dxr*::*kan* cannot grow without the expression of Dxr_CT_ *in trans*, these data indicate that Dxr_CT_ functions similarly to Dxr_EC_.

**Table 1:** *Escherichia coli* strains and plasmids used in this study.

Strain or Plasmid	Characteristics^a^	Source
**Strains**		
**MG1655**	F factor-cured derivative of W1485 via acridine orange curing. Suppressor negative; reference strain for genome sequence of *E*. *coli* K-12.	[28]
**ATM1442**	*E*. *coli* MG1655 pBAD24::*dxr*_EC_; Amp^R^	This study
**ATM1443**	*E*. *coli* MG1655 pBAD24::*dxr*_CT_; Amp^R^	This study
**ATM1469**	*E*. *coli* MG1655 pBAD24; Amp^R^	This study
**ATM1470**	ATM1443 Δ*dxr*::*kan*; Amp^R^, Kan^R^	This study
**ATM1471**	ATM1442 Δ*dxr*::*kan*; Amp^R^, Kan^R^	This study
**Plasmids**		
**pBAD24**	arabinose inducible/glucose suppressible expression plasmid; Amp^R^	[29]
**pSIM9**	lambda Red expression vector expressing *exo*, *bet*, and *gam* under the control of the lambda repressor; Cm^R^	[30]
**pJAS5**	pBAD24::*dxr*_EC_; Amp^R^	This study
**pJAS6**	pBAD24::*dxr*_CT_; Amp^R^	This study

^a^ Amp^R^, ampicillin resistance; Kan^R^, kanamycin resistance; Cm^R^, chloramphenicol resistance.

### Overexpression of Dxr_CT_ in *E*. *coli* reverses FSM inhibition

**Fig 4:**
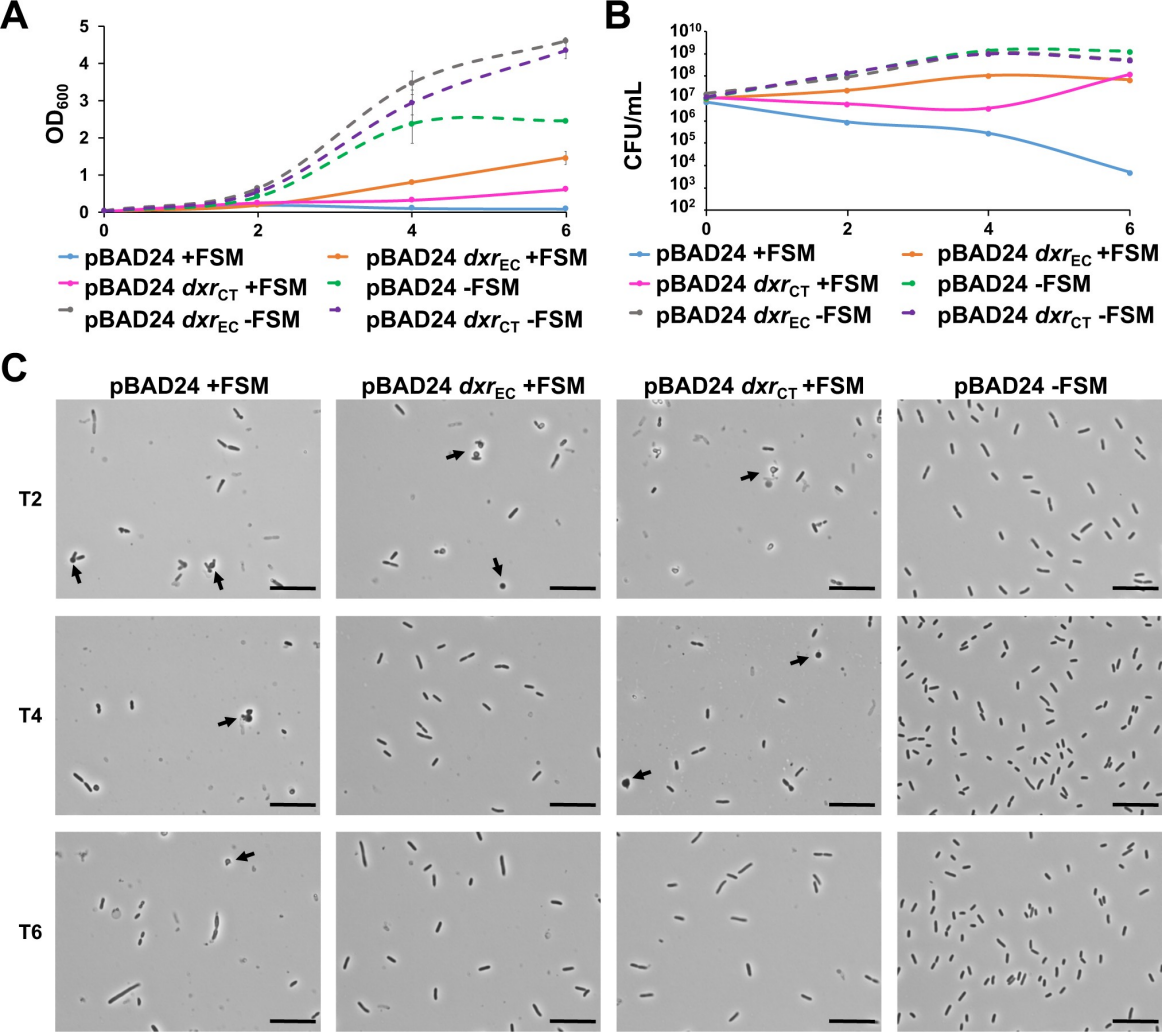
Overexpression of Dxr_CT_ in *E*. *coli* supports growth and cellular morphology under FSM exposure. Overnight cultures of *E*. *coli* MG1655 carrying the indicated plasmids were subcultured 1:100 in LB broth + 0.2% arabinose either without FSM, or with 25 μM FSM. OD_600_ (A) and CFU (B) were determined every two hours for 6 hours. Phase contrast microscopy (C) was performed every two hours from T2 to T6. Arrows indicate rounded cell morphology observed during FSM exposure. Scale bars represent 10 μm. OD_600_ and microscopy data (A and C) are representative of three independent experiments, each performed with duplicate cultures. For CFU data (B) each curve depicts a single replicate that is representative of two independent experiments preformed in duplicate. Error bars in panel A represent the average of duplicate cultures from a single experiment +/- SD.

Because Dxr is a known target for FSM, we sought to determine whether overexpression of Dxr_CT_ could rescue the growth of *E*. *coli* K-12 MG1655 exposed to inhibitory concentrations of FSM. To measure susceptibility to FSM, optical density at 600 nm (OD_600_) and colony forming units (CFU) were determined at 0, 2, 4 and 6 hours post inoculation (hpi) (**Fig 4A and 4B**). Cultures transformed with the empty pBAD24 vector (ATM1469) grew normally in the absence of FSM but did not grow when exposed to 25 μM FSM for 6 hours. However, by 6 hpi, FSM-treated cultures overexpressing either Dxr_EC_ (ATM1442) or Dxr_CT_ (ATM1443) exhibited elevated OD_600_ values and increased viable counts compared to the FSM-treated empty vector control **(Fig 4A and 4B)**.

While FSM-treated cultures overexpressing Dxr_CT_ and Dxr_EC_ exhibited similar viable counts by 6 hpi, the OD_600_ values for cultures overexpressing Dxr_CT_ were lower compared to cultures overexpressing Dxr_EC_. When *E*. *coli* are exposed to FSM, spherical cells and cell lysis have been observed beginning around 2 hours post treatment [31]. Therefore, we examined the effect of FSM treatment on bacterial cell morphology using phase contrast microscopy **(Fig 4C)**. Untreated vector only control cells exhibited normal rod shapes and increased in cell density over time, which was consistent with the OD_600_ and CFU data. At 2 hpi cell density in all FSM-treated cultures was lower compared to the untreated control and all FSM-treated cultures contained spherical cells among normal rod-shaped cells. By 4 hpi, cell density continued to decrease in FSM-treated, empty vector control cultures and few rod-shaped cells were observed. By 6 hpi, very few cells were observed in the FSM-treated empty vector control culture. In FSM-treated cultures overexpressing Dxr_EC_, most cells were rod-shaped and very few spherical cells were observed by 4 hpi. By 6 hpi, no spherical cells were observed. Cells overexpressing Dxr_CT_ exhibited spherical, aggregated cells through 4 hpi and cells appeared shorter at 2 and 4 hpi compared to Dxr_EC_ overexpressing cells. By 6 hpi, cells overexpressing Dxr_CT_ had mostly returned to rod shapes.

The observation of shorter cells and spherical cells for a longer duration in cultures overexpressing Dxr_CT_ may explain why the CFU were comparable to cultures overexpressing Dxr_EC_ by 6 hpi despite the lower optical density readings in cultures overexpressing Dxr_CT_. Although Dxr_CT_ is somewhat less efficient in restoring normal cell morphology in *E*. *coli*, our data support the hypothesis that Dxr_CT_ functions similarly to Dxr_EC_ because overexpression of Dxr_CT_ can effectively overcome the growth inhibitory effects of FSM on *E*. *coli*.

### *C*. *trachomatis* developmental cycle is inhibited by FSM in HeLa cell culture

**Fig 5:**
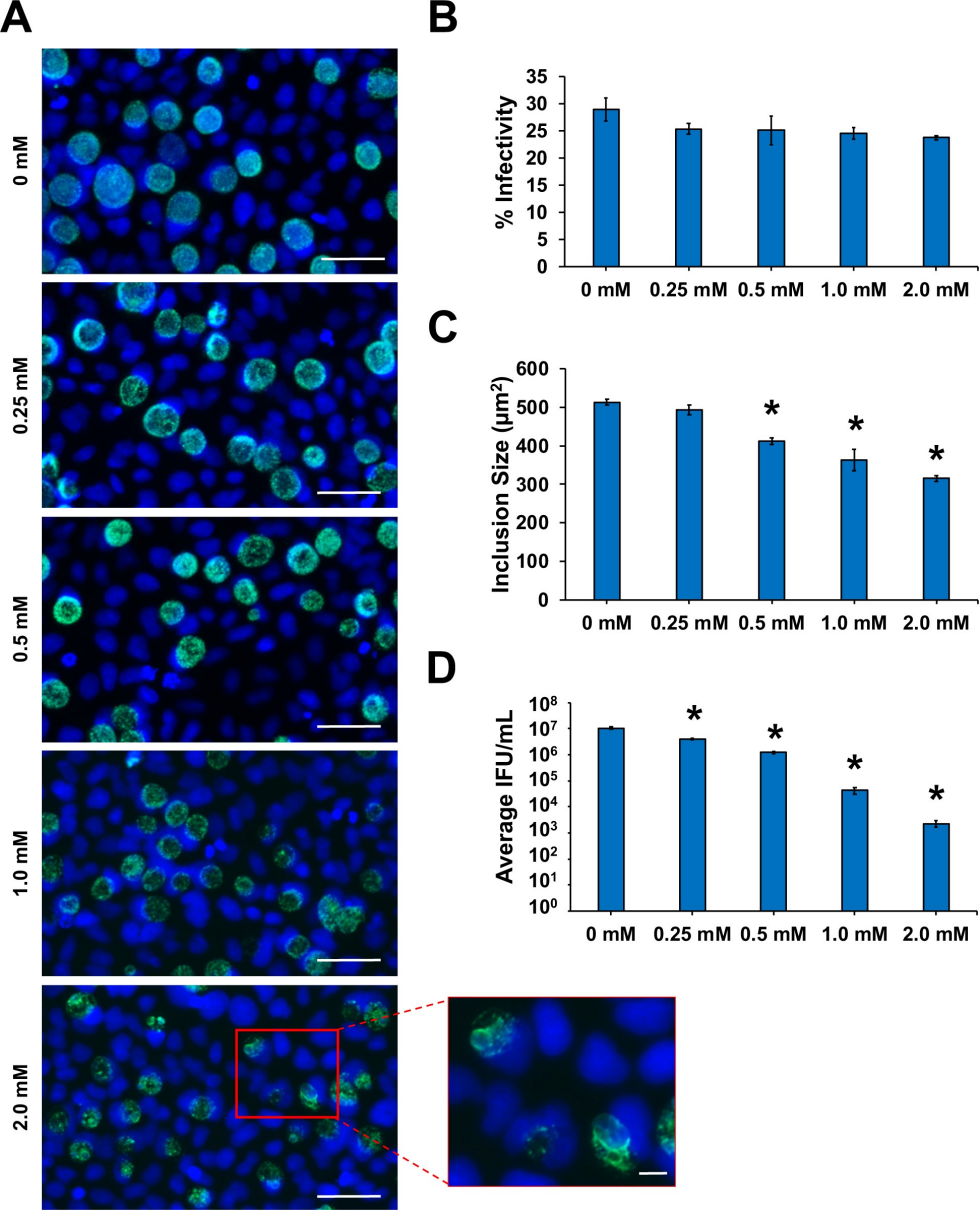
Chlamydial inclusion size and production of infectious EB are significantly inhibited by FSM exposure. HeLa cells were infected with *C*. *trachomatis* at an MOI of 0.5 and harvested for analysis at 40 hpci. (A) Representative 400X magnification fluorescence microscopy images of chlamydial inclusions (green) stained with BioRad anti-chlamydial LPS Pathfinder stain and HeLa cell nuclei (blue) counter-stained with DAPI. Scale bars = 50 μm. Insert from 2 mM FSM concentration depicts zoomed-in view of ABs. Scale bars = 10 μm. (B) Percent infectivity was calculated by counting the number of inclusions/cell nuclei per field in 10 fields per coverslip in triplicate samples. (C) The area of 150 random inclusions per triplicate sample were measured using the spline contour tool in the Zen Blue Zeiss software package. (D) Production of infectious EBs was determined via chlamydial titer assays by subpassage. Significant (p ≤ 0.005) difference from untreated samples is indicated by an asterisk (*). Error bars indicate +/- SEM from triplicate samples and data are representative of three independent experiments.

*Chlamydia* susceptibility to FSM has not been previously examined. Treatment of A549 lung cancer cells and RAW264.7 macrophage cells infected with the facultative intracellular bacterial pathogen, *Francisella novicida*, with 0.25 mM FSM results in roughly 98% inhibition of bacterial growth in both cell types [19]. Early in infection, *Francisella* species initially reside within host phagosomes, but escape to replicate freely in the host cell cytosol four hours after infection [32, 33]. Because *Chlamydia* replicates within a membrane bound inclusion in the host cell cytosol, we predicted that higher concentrations of FSM might be necessary to observe any effect on growth. Therefore, we initially exposed *C*. *trachomatis*-infected HeLa cell cultures to 0.25 and 0.5 mM FSM. Immunofluorescence assays (IFAs) performed at 40 hpci (hours post chlamydial infection) demonstrated that exposure to FSM did not affect percent infectivity at either concentration, but exposure to 0.5 mM FSM resulted in significantly decreased inclusion size in infected cultures (**Fig 5A–5C**). Compared to untreated samples, 0.25 and 0.5 mM FSM significantly reduced chlamydial titer (**Fig 5D**), but chlamydial titer did not decrease lower than the initial infection inoculum (1.3 x 10^5^ IFU), indicating that these concentrations of FSM were still permissive for chlamydial replication. Therefore, concentrations >0.5 mM FSM were tested. Exposure to 2 mM FSM altered chlamydial morphology, as many AB-like forms were observed (**Fig 5A**). After 40 h exposure to 2 mM FSM, titers were not only significantly reduced compared to the untreated control but were also reduced compared to the initial 1.3 x 10^5^ IFU inoculum (**Fig 5D**). Importantly, 2 mM FSM did not affect chlamydial infectivity (**Fig 5B**) but did reduce inclusion size compared with 0.25, 0.5 mM, and 1.0 mM FSM (**Fig 5C**). Furthermore, the highest concentration of FSM we tested (3 mM) showed no toxicity to HeLa cell cultures (**S2 Fig**). Altogether, these data demonstrate that FSM does not inhibit the ability of *Chlamydia* to infect HeLa cells but prevents optimal inclusion development and production of infectious progeny, suggestive of a persistent state.

### FSM treatment induces persistence in *C*. *trachomatis*

**Fig 6:**
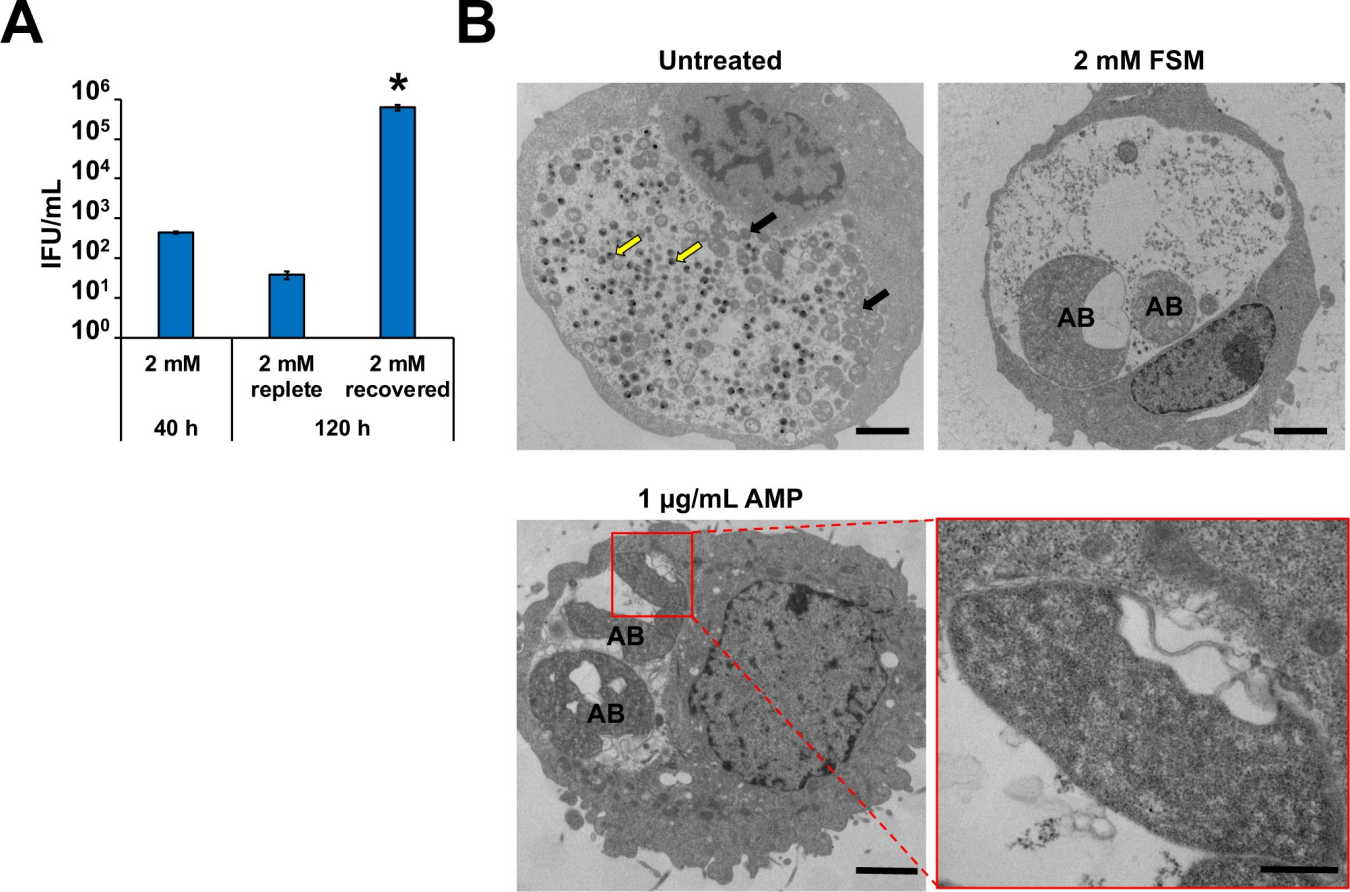
FSM induces persistence in *Chlamydia*-infected HeLa cell cultures. At 40 hpci, cultures were washed once with complete media and either re-fed with media containing FSM (replete) or without FSM (recovered). Samples were incubated an additional 80 h and titers (A) were examined at 120 hpci. Asterisk (*) indicates significant difference from 120 h, 2 mM replete samples. Error bars indicate +/- SEM from triplicate samples and data are representative of three independent experiments. (B) Electron micrographs from untreated and 2 mM FSM-treated *Chlamydia*-infected HeLa cells 40 hpci. Yellow arrows and black arrows indicate normal EBs and RBs, respectively. AB indicates aberrant bodies. Scale bars = 2 μm. For AMP insert (lower right panel), scale bars = 500 μm.

Due to the possibility that FSM prevents the synthesis of bactoprenol (required for peptidoglycan synthesis) in *Chlamydia* and that the chlamydiae appear to be aberrant when exposed to 2 mM FSM in culture (**Fig 5A**), we examined whether FSM induces persistence in *Chlamydia*. Chlamydial titer is significantly reduced in the presence of β-lactam antibiotics, but if cultures are washed and β-lactam antibiotic-free media is added, *Chlamydia* can re-enter and complete normal development, leading to restoration of chlamydial EB production [12]. To determine whether the reduction in infectious titer was due to entry of *Chlamydia* into persistence, we first examined whether chlamydial EB production can be restored in FSM-treated cultures. Cultures exposed to 2 mM FSM for 40 h produced very few EBs (**Fig 6A).** After 40 h, parallel cultures were washed once with FSM-free media, re-fed with either FSM-replete or FSM-free media, and then incubated for an additional 80 h. Cells continuously exposed to 2 mM FSM for 120 h displayed titers similar to cultures grown for 40 h at the same FSM concentration. However, FSM treated cultures washed and re-fed with FSM-free media at 40 hpci (and incubated an additional 80 h) demonstrated significantly elevated titers compared to cultures maintained with FSM (**Fig 6A**). To confirm that FSM induces AB formation, chlamydial morphology was examined using electron microscopy. Compared to untreated cultures, *Chlamydia*-infected cultures exposed to 2 mM FSM for 40 h exhibited enlarged morphology consistent with ABs (**Fig 6B**) [13]. Furthermore, FSM-treated ABs exhibit membrane gaps similar to those observed with β-lactam antibiotic treatment (**Fig 6B**) [34].Thus, FSM exposure induces persistence in *C*. *trachomatis*.

### FSM treatment prevents normal peptidoglycan ring formation in *C*. *trachomatis*

**Fig 7:**
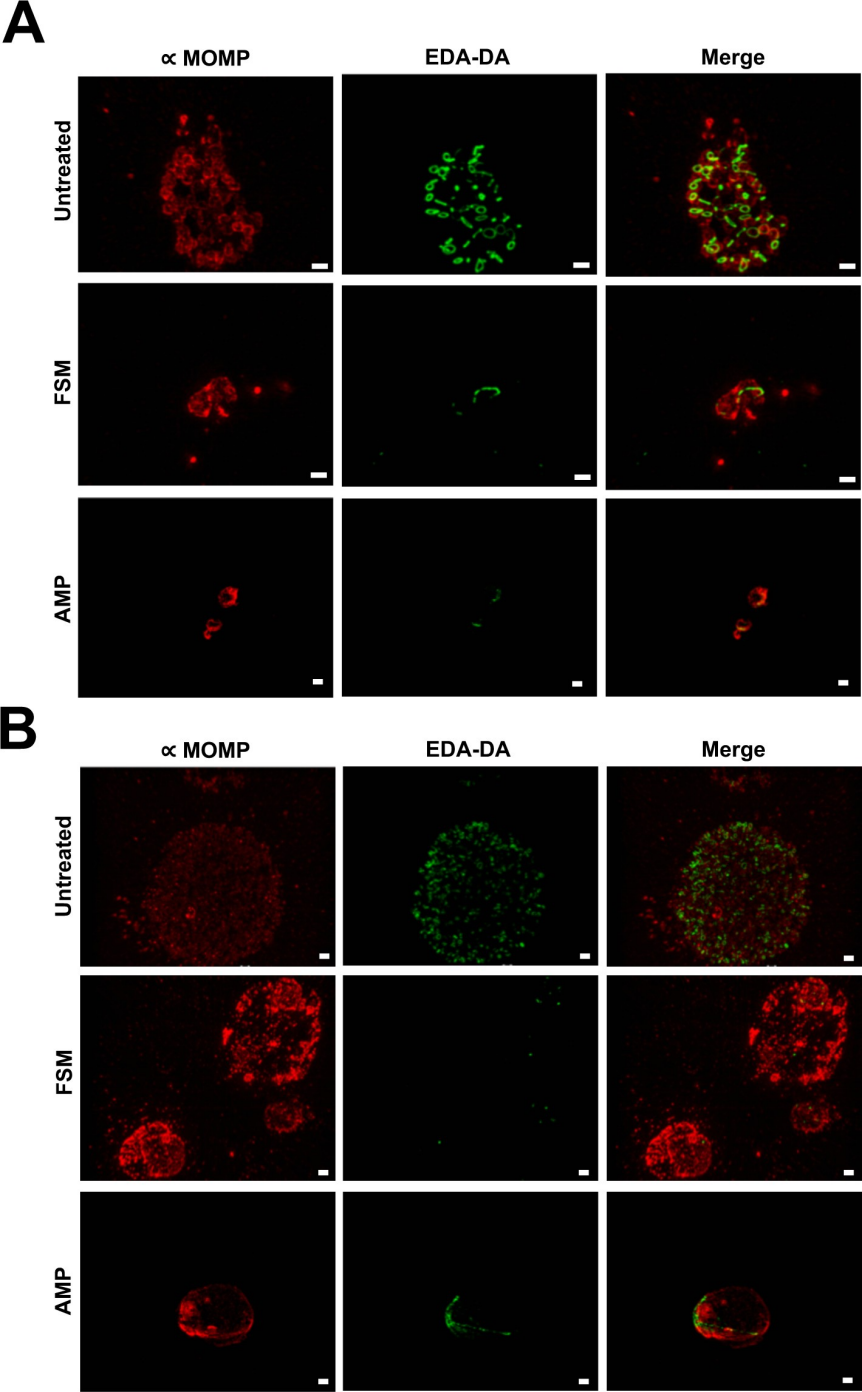
Structured illumination microscopy of D-amino acid dipeptide (DAAD) peptidoglycan labeling in FSM-treated *C*. *trachomatis*. Structured illumination microscopy was conducted on untreated, FSM-treated, or AMP-treated *C*. *trachomatis-*infected HeLa cells. 1 mM EDA-DA and either 1 μg/mL AMP or 2.44 mM FSM were added at T0 and coverslips were fixed at 14 h (A) or 38 h (B) post chlamydial infection. Peptidoglycan labeling (represented by EDA—DA) is shown in green and the *C*. *trachomatis* major outer membrane protein (MOMP) is shown in red. Images are representative of ~20 inclusions viewed per condition. Scale bars = 1 μm.

In most Gram negative bacteria, peptidoglycan is continuously detectable throughout bacterial growth and forms a structure known as a sacculus that encases each bacterium [35]. In *Chlamydia*, peptidoglycan does not form a sacculus, but appears as a ring-like structure only in RBs [36–38]. Since peptidoglycan is not detectable in EBs, peptidoglycan is thought to be required for chlamydial cell division, which occurs during the RB phase of development [36, 37, 39]. Under exposure to the β-lactam antibiotic ampicillin, peptidoglycan structure is altered and exhibits filamentous-like labeling resembling broken rings (**Fig 7**) [37]. *Chlamydia*-infected cultures exposed to 2.44 mM FSM and the peptidoglycan labeling probe, ethynyl-D-alanyl-D-alanine (EDA-DA) for 14 h demonstrate a similar labeling pattern to β-lactam exposed cultures (**Fig 7A**). Furthermore, very little peptidoglycan labeling is detectable in the FSM treated inclusions after 38 h (**Fig 7B**). Together, these data indicate that FSM-induced inhibition of isoprenoid synthesis prevents peptidoglycan synthesis in *Chlamydia*.

## Discussion

A number of stimuli can induce persistence in *C*. *trachomatis* including interferon-y exposure, β-lactam antibiotic exposure, nutrient deprivation, and viral co-infection [12, 40–43]. All of these persistence inducers cause reversible AB formation and reductions in infectious progeny production [11]. Here, we describe FSM antibiotic exposure as a novel persistence inducer that operates by inhibiting Dxr and MEP pathway-dependent isoprenoid synthesis. In the case of FSM-induced persistence, we observed significantly reduced infectious progeny production that is restored upon removal of FSM from the tissue culture media. FSM exposure also resulted in AB formation and abnormal peptidoglycan ring formation.

Peptidoglycan ring labeling in *Chlamydia* ABs was previously performed for β-lactam antibiotic-exposed cultures and for chlamydial cultures exposed to inhibitors that prevent the activity of the bacterial actin-like cytoskeletal protein, MreB [36, 37]. In *Chlamydia*, MreB is thought to play a central role in cell division by directing peptidoglycan ring localization to the division septum [36]. When exposed to MreB inhibitors, slightly enlarged ABs are observed. Furthermore, the peptidoglycan ring dissociates and peptidoglycan becomes undetectable [36]. Like other persistence phenotypes, MreB AB formation is reversible upon removal of MreB inhibitors from the culture medium and peptidoglycan ring detection is restored [36]. However, FSM-treated *Chlamydia* cultures exhibited detectable but abnormal peptidoglycan ring structures, similar to the labeling pattern observed during β-lactam antibiotic exposure (**Fig 7**) [37]. β-lactam antibiotics target the penicillin-binding proteins (PBPs) that reside in the periplasm and function in crosslinking peptidoglycan monomers. Transglycosylation remains unaffected, resulting in detection of “broken” peptidoglycan rings [36]. In addition to the similarities between peptidoglycan ring formation during FSM-exposure and β-lactam antibiotic exposure, electron micrographs from β-lactam antibiotic exposed ABs exhibit large membrane gaps similar to the membrane gaps we observed after exposure to FSM (**Fig 6B**) [34]. While electron microscopy has not been performed for *Chlamydia* exposed to MreB inhibitors, we predict that inhibition of isoprenoid synthesis likely does not induce rapid peptidoglycan degradation as in the case of MreB inhibition. We propose that FSM prevents peptidoglycan synthesis in *Chlamydia* that leads to persistence induction by limiting availability of the key isoprenoid, bactoprenol.

Bactoprenol, also known as undecaprenyl phosphate, is a 55-carbon isoprenoid with two critical functions: lipopolysaccharide (LPS) synthesis and peptidoglycan synthesis [44]. Bactoprenol functions as an anchor in the inner leaflet of the inner membrane for the synthesis of the O-antigen region of LPS. Rather than making a traditional LPS, *Chlamydia* produces lipooligosaccharide (LOS), which lacks O-antigen. Because chlamydial LOS does not contain O-antigen, its synthesis does not require bactoprenol and is unaffected by FSM as seen by the ability to visualize FSM-treated *Chlamydia*-infected cultures with a stain targeting chlamydial LOS. However, bactoprenol also plays a central role in the synthesis of the major bacterial cell wall constituent, peptidoglycan. Peptidoglycan regulates osmotic pressure, serves as an anchor for a multitude of bacterial proteins, and is critical to cell division [45]. Peptidoglycan synthesis is a multistep process that begins with precursor assembly in the bacterial cytoplasm. The final steps in precursor assembly are mediated by the lipid carrier, bactoprenol, which resides within the inner leaflet of the inner membrane. Assembled precursors called lipid II are then flipped across the inner membrane from the cytoplasm to the periplasm and are incorporated into nascent peptidoglycan (**Fig 1 steps 1–3**) [46]. Bactoprenol production is essential for lipid I and lipid II synthesis and efficient trafficking of newly synthesized peptidoglycan precursors to the bacterial periplasm [47]. We predict that isoprenoid pools are significantly depleted as a result of FSM treatment, resulting in aberrant body formation. As chlamydial RBs require peptidoglycan in order to complete cell division, the loss of isoprenoid precursors necessary to generate bactoprenol, and subsequently the lipid II needed to replace peptidoglycan degraded during septation, results in the loss of the peptidoglycan ring and the complete inhibition of cell division.

While our data show that FSM exposure induces persistence in *Chlamydia*, a previous study by Grieshaber et al demonstrated that metabolites generated by IspE, the fourth enzyme in the MEP pathway, were required for the dissociation of the chlamydial histone H1-like protein (Hc1) from DNA *in vitro* [25]. Since dissociation of Hc1 is likely required for EB to RB conversion, it seems possible that rather than preventing cell division, FSM exposure would prevent this early event in development. All seven chlamydial MEP pathway genes are transcribed starting at 2 hpci [25]. Therefore, some isoprenoid precursors are likely synthesized prior to Dxr inhibition by FSM, allowing for EB to RB conversion. We attempted to prevent EB to RB conversion by pre-loading HeLa cells with increasing concentrations of FSM and maintaining continuous exposure to FSM during the 2 h adsorption period and 40 h infection (S3 Fig). Pre-loading HeLa cells with 1.0 mM FSM resulted in EB to RB transition and formation of aberrant RBs similar to those observed for non-pre-treated cultures exposed to 2 mM FSM. Since preloading the host cells with FSM was not effective at preventing AB development, we conclude that FSM does not inhibit EB to RB conversion.

In the MEP pathway, two iron-sulfur cluster-containing enzymes, IspG and IspH, are downstream from Dxr. Upon entry into the bacterial cytoplasm, free iron must be assembled into iron-sulfur clusters and chaperoned to the enzymes that require these cofactors to function [48]. We postulate that *Chlamydia* encountering unfavorably low environmental iron levels become depleted of iron necessary for iron-sulfur cluster assembly and enter persistence, as these critical co-factors are no longer available for delivery to IspG/IspH. In this manner, synthesis of isoprenoid precursors would cease, preventing the production of a critical isoprenoid, bactoprenol. Thus, it is reasonable to speculate that inhibition of isoprenoid synthesis contributes, at least in part, to the persistence mechanism induced by iron deprivation.

In conclusion, the use of FSM to inhibit isoprenoid synthesis by targeting the MEP pathway, and specifically Dxr, has revealed that chlamydial Dxr functions similarly to *E*. *coli* Dxr and that the MEP pathway is functional in *Chlamydia*. More importantly, we demonstrate that FSM is a novel inducer of chlamydial persistence. Like other known persistence inducers, inhibition of isoprenoid synthesis results in AB formation and significantly decreased EB production [12, 43, 49, 50]. Furthermore, our data demonstrate similarities in abnormal peptidoglycan ring formation previously observed after *Chlamydia* is exposed to the peptidoglycan synthesis-targeting antibiotic, ampicillin [37]. The isoprenoid bactoprenol is critical for peptidoglycan synthesis. Therefore, we conclude that inhibition of isoprenoid synthesis by FSM exposure reduces isoprenoid precursor production and bactoprenol availability. In this regard, targeted inhibition of isoprenoid synthesis has revealed that isoprenoids are critical molecules required for cell division in *Chlamydia*.

## Methods

### Cells and bacteria used in this study

HeLa-USU cervical carcinoma cells (a variant of HeLa-CCL2 from the American Type Culture Collection) were used in all tissue culture assays [51]. *E*. *coli* DH5α and wild type *E*. *coli* K-12 strain MG1655 [28, 52] were used for cloning and growth assays, respectively. *C*. *trachomatis* L2 434/Bu was provided by Harlan Caldwell (Rocky Mountain Laboratories, Hamilton, MT) and used for cloning and all infection experiments.

### Structural modeling of *C*. *trachomatis* Dxr

The web-based server, Phyre2, was used to predict the three-dimensional (3D) model for *C*. *trachomatis* L2 Dxr. Phyre2 uses homology detection to predict a 3D structure, ligand-binding site and the effect of amino acid variants for the given sequence [53]. To verify the binding pocket of the predicted 3D structure of *C*. *trachomatis* Dxr, the UCSF Chimera package was used [54]. The Protein Data Base structure of *E*. *coli* Dxr (1Q0L) served as the reference. The predicted 3D structure of *C*. *trachomatis* Dxr was superimposed on the *E*. *coli* Dxr reference structure and the FSM binding pocket was analyzed.

### *dxr* Cloning, Growth Curves, and Phase Contrast Microscopy

*dxr* was amplified from *E*. *coli* K-12 MG1655 and *C*. *trachomatis* L2 genomic DNA using primers specified in **S1 Table**. The products were ligated into the pBAD24 arabinose inducible/glucose suppressible vector [55], creating pJAS5 and pJAS6, respectively. The empty vectors, pJAS5, and pJAS6 were transformed into *E*. *coli* K-12 MG1655 for overexpression studies. Overnight cultures were diluted 1:100 in 20 mL duplicate cultures of Luria-Bertani (LB) broth (BD Difco, Franklin Lakes, NJ) containing 100 μg/mL ampicillin. At time 0, 25 μM FSM (InvivoGen, San Diego, CA) and 0.2% arabinose were added. Optical Density was measured at 600 nm (OD_600_) every two hours for 6 hours using a Biomate 3S spectrophotometer (Thermofisher). In addition to OD_600_, colony forming units (CFU) were determined at each time point to assess bacterial viability. At 2, 4, and 6 hours post inoculation (hpi), 500 μL of culture was concentrated via centrifugation to increase the number of bacteria visible per field. At each time point, the FSM-treated vector only control was 5X concentrated. At 2 hpi, untreated and FSM-treated samples overexpressing Dxr_EC_ or Dxr_CT_ were 5X concentrated; at 4 hpi and 6 hpi these samples were 2X concentrated. Ten μL of each bacterial culture was examined by phase contrast microscopy under a 100X oil immersion objective and an Olympus BX50 light and fluorescence microscope with accompanying DP Controller imaging software.

### *E*. *coli dxr* Conditionally Lethal Mutants

*E*. *coli* K-12 MG1655 previously transformed with pBAD24::*dxr*_CT_ (ATM1443) was transformed with pSIM9. Lambda red recombination was performed as previously described [56]. Briefly, overnight LB cultures grown at 30°C with 0.2% arabinose and 100 μg/ml ampicillin were subcultured the next day under the same conditions and grown to an OD_600_ between 0.4 and 0.6. Induction of the pSIM9 λ-Red recombinase genes proceeded at 42°C with shaking for 15 min while uninduced cultures were maintained at 30°C. Cultures were cooled on ice for 5 min then prepared for electroporation of the *dxr*::*kan* linear fragment. The *dxr*::*kan* linear fragment was amplified with primers listed in **S1 Table**. The fragment contains upstream and downstream *dxr* sequences that flank a kanamycin resistance cassette amplified using pKD4 as the template. After electroporation of *dxr*::*kan*, recovery proceeded for 2 hrs at 30°C with shaking and cultures were plated on LB agar plus 0.5% arabinose, 100 μg/mL ampicillin, and 50 μg/mL kanamycin. Colonies were screened for allelic exchange using a forward primer upstream of the *dxr* start site and a reverse primer recognizing the *kan* cassette (**S1 Table**) and passaged twice at 37°C in the absence of chloramphenicol to segregate out pSIM9. The Δ*dxr*::*kan* mutation was transduced into a clean background of MG1655-pBAD24::*dxr*_EC_ and colonies were similarly screened.

To demonstrate that Dxr_CT_ supports the growth of an *E*. *coli* Dxr conditional mutant, duplicate cultures of MG1655 Δ*dxr*::*kan* pBAD24::*dxr*_EC_ (ATM1442) and MG1655 Δ*dxr*::*kan* pBAD24::*dxr*_CT_ (ATM1443) were inoculated into 3 mL LB cultures containing 0.2% arabinose. The next day, 5 μL of each culture were streaked for isolation on plates containing 100 μg/mL ampicillin, 50 μg/mL kanamycin and either 0.5% arabinose or 0.5% glucose. Plates were incubated overnight and photographed the next morning using a Chemidoc Imaging System (BioRad, Hercules, CA).

### Chlamydial infection and recovery assays

HeLa cells were plated at 1.3 x 10^5^ cells/mL in triplicate wells of 24 well plates either with or without glass coverslips for use in immunofluorescence assays (IFA) and titer assays, respectively. After 24 h, monolayers were infected with a multiplicity of infection of 0.5 *C*. *trachomatis* L2 in 100 μL sucrose-phosphate-glutamic acid buffer per well. Cultures were incubated at 37°C for 2 hours with agitation every 30 minutes to allow for adsorption. At T0, inocula were removed and cultures were re-fed with Dulbecco’s Modified Eagle’s Medium (DMEM) +10% FBS, 1 μg/mL cycloheximide. FSM was added at T0. At 40 h post infection, cultures seeded on coverslips were fixed with 0.5 mL cold methanol for 20–30 min and stored in wash buffer for staining. After 40 h, infected monolayers lacking coverslips were scraped using sterile 200 μL large orifice pipette tips, collected in the pre-existing 1 mL of culture media and immediately frozen at -80°C for later use in *Chlamydia* titer assays. To pre-load HeLa cells with FSM, increasing concentrations of FSM were added during cell plating, 24 h prior to infection. The FSM concentration was maintained during the 2 h adsorption period and throughout the 40 h infection, at which time samples were similarly processed for IFA and titer assays.

In FSM recovery assays, parallel infected wells were exposed to 2 mM FSM for 40 h, then washed once with DMEM +10% FBS. Triplicate samples were either re-fed with media containing 2 mM FSM (replete) or with antibiotic-free media (recovered). Washed and re-fed cultures were incubated at 37°C for an additional 80 h. At 120 hpci, IFA samples and titer samples were harvested.

### Percent Infectivity and inclusion size measurement immunofluorescence assays

Percent infectivity and inclusion size measurements were performed similarly as previously described [57]. Briefly, fixed coverslips were stained with Pathfinder *Chlamydia* LPS stain (BioRad, Hercules, CA) to visualize chlamydial inclusions and were counterstained with DAPI to visualize host cell nuclei. Ten fields were captured for each coverslip under 400X magnification using a Z1 Axiovert Observer Epifluorescence microscope (Zeiss, Oberkochen, Germany). Using the accompanying Zen Blue software, a grid was applied to the photographs to exclude nuclei and inclusions on the edges of frames. Nuclei and inclusions were counted and percent infectivity was determined by dividing the number of inclusions by the number of nuclei and multiplying by 100. Data are expressed as the average percent infectivity of three coverslips per sample +/- SEM. To determine inclusion size, five of the ten images were analyzed per coverslip. The spline contour tool was used to circle 10 inclusions per field. The area of 150 total inclusions was measured per sample. Data are expressed as the average inclusion size in μm^2^ +/- SEM.

### Chlamydial Titer by Subpassage

To determine the production of infectious progeny, titer samples were thawed, vortexed and sonicated and then serially diluted for infection of a new monolayer of HeLa cells. After 40 h, coverslips were fixed with methanol and stained as in immunofluorescence assays to visualize chlamydial inclusions [57]. Inclusions were counted on three coverslips per sample using 200X magnification and a Zeiss Z1 Axio Observer inverted light and epifluorescence microscope. Data are expressed as the average inclusion forming units/mL +/- SEM.

### MTT assay

HeLa cells were plated in DMEM + 10% FBS at a density of 5 x 10^4^ cells/0.2 mL per well in 96 well microtiter plates. After 24 h, media was removed and replaced with media containing 0.25, 0.5, 1.0, 2.0, 2.44, and 3.0 mM FSM and 1 μg/mL cycloheximide. Plates were incubated for 40 h at 37°C with 5.0% CO_2_. At 40 h, the media containing FSM was replaced with DMEM lacking phenol red and the MTT assay was performed according to manufacturer’s instructions (Cayman, MI, USA). Viability was determined from triplicate wells and is represented as the average OD_570_ +/- SD.

### Transmission electron microscopy

Untreated and 2 mM FSM-treated samples were harvested at 40 hpci and prepared for transmission electron microscopy. *Chlamydia*-infected HeLa cells treated with 1 μg/mL ampicillin (AMP) (Sigma-Aldrich, MO, USA) were harvested at 22 hpci. Pelleted bacterial samples were suspended for 1 h at room temperature in a solution of 2% formaldehyde (freshly prepared from paraformaldehyde crystals) and 2% EM grade glutaraldehyde (Electron Microscopy Sciences, Hatfield, PA) prepared in 0.1 M cacodylate buffer, pH 7.2. Following three washes in 0.1 M cacodylate buffer, samples were incubated for 10 minutes in 2% agarose LE (Electron Microscopy Sciences, Hatfield, PA) at 40°C. Samples were then incubated at 4°C for five minutes. Once solidified, the agarose embedded samples were removed from the sample tubes for subsequent processing. Samples were incubated in a 2% OsO_4_ solution prepared in 0.1 M cacodylate buffer for one hour and washed 3 x 10 min in 0.1 M cacodylate buffer. Samples were then dehydrated in a graduated series of ethanol (1 x 10 min each in 30%, 50%, 70%, and 95% ethanol and 2 x 10 min in 100% ethanol). Following dehydration, samples were infiltrated in a graduated series of Spurr's epoxy resin (Electron Microscopy Sciences, Hatfield, PA) and polymerized at 70°C for 11 h. Polymerized blocks were sectioned in a Leica UC6 ultramicrotome and thin sections were collected on 3 mm copper grids. Grids were post-stained in a Leica EM AC20 and examined on a JEOL JEM-1011 transmission electron microscope (JEOL USA, Peabody, MA). Images were collected on an Advanced Microscopy Techniques digital camera (AMT Corp., Woburn, MA).

### Peptidoglycan labeling

Peptidoglycan labeling and chlamydial staining were performed as previously described [36, 37]. 1 mM EDA-DA probe and 2.44 mM FSM or 1 μg/mL AMP were added at T0 after the 2-hour adsorption period. Click chemistry and staining of *Chlamydia* major outer membrane protein (MOMP) was performed at 14 h and 38 h post chlamydial infection. Structured illumination microscopy (SIM) was conducted to visualize peptidoglycan ring formation as described [36, 37].

### Statistical analysis

Differences in infectivity, inclusion size, and chlamydial titer assays between untreated and FSM treated samples were determined using the unpaired Student’s t-test. Values of p ≤0.05 are considered significant and are indicated by an asterisk (*).

## Supporting information

S1 Fig The 3D alignment of the Dxr_EC_ crystal structure with the putative Dxr_CT_ structure. (PDF)

S2 Fig Up to 3 mM FSM is not toxic to HeLa cell cultures. HeLa cells were incubated with increasing concentrations of FSM for 40 h and viability was assessed using the MTT assay. Data are representative of the average of triplicate samples from one experiment. 10% SDS was used as a negative control for viability. Error bars indicate +/- SD and significant (p ≤ 0.005) difference from untreated samples is indicated by an asterisk (*).
(TIF)

S3 Fig Pre-loading HeLa cells with FSM does not prevent EB to RB conversion. For pre-loaded samples (pre) FSM was added to tissue culture media at the time of cell plating (24 h prior to infection) and was maintained during 2 h adsorption and throughout the 40 h incubation. HeLa cells with and without FSM pre-treatment were infected with *C*. *trachomatis* at an MOI of 0.5, exposed to the indicated FSM concentrations and harvested for analysis at 40 hpi. (A) Representative 400X magnification fluorescence microscopy images of chlamydial inclusions (green) stained with BioRad anti-chlamydial LPS Pathfinder stain and HeLa cell nuclei (blue) counter-stained with DAPI. Yellow arrows indicate examples of aberrant RBs. Scale bars = 50 μm. (B) Percent infectivity was calculated by counting the number of inclusions/cell nuclei per field in 10 fields per coverslip in triplicate samples. (C) The area of 150 random inclusions per triplicate sample were measured using the spline contour tool in the Zen Blue Zeiss software package. (D) Production of infectious EBs was determined via chlamydial titer assays by subpassage. Significant (p ≤ 0.005) difference from untreated samples is indicated by an asterisk (*). Error bars indicate +/- SEM from triplicate samples and data are representative of two independent experiments.
(TIF)

S1 Table Primers used in this study. (DOCX)
