# Supplementary material for: Fosmidomycin, an inhibitor of isoprenoid synthesis, induces persistence in Chlamydia by inhibiting peptidoglycan assembly
Source: PLoS Pathog. 2019 Oct 17;15(10):e1008078. doi: 10.1371/journal.ppat.1008078 (PMC6818789; doi:10.1371/journal.ppat.1008078)
Supplement: S1 Fig — (PDF) [file ppat.1008078.s001.pdf]

```
                   1          11         21         31         41
Consensus            MKqLaiiGST GSIGcqtLqV VRhiPehFii eaLaAGkNqe amisQirEFs
Conservation
Ctl2_dxr_.pdb      1 MKHLALIGST GSIGRQVLQV VRSIPDTFII ETLAAGRNQE ALISQIREFN
E_coli_dxr.pdb     1 MKQLTILGST GSIGCSTLDV VRHNPEHFRV VALVAGKNVT RMVEQCLEFS

                   51         61         71         81         91
Consensus            PRyaamreEa syKeLkkml. ...phiEiLs GqqaacsmAa epsVtitiaA
Conservation
Ctl2_dxr_.pdb     51 PRVVAVREET TYKELRKLF. ...PHIEILL GEEGLVSVAT EPSVTITIVA
E_coli_dxr.pdb    51 PRYAVMDDEA SAKLLKTMLQ QQGSRTEVLS GQQAACDMAA LEDVDQVMAA

                   101        111        121        131        141
Consensus            isGiaaLpat iAAIRqkKTI aLANKESLVa aGrLfmtaak qskaQiLPiD
Conservation
Ctl2_dxr_.pdb     97 SSGIDALPAV IAAIRQKKTI ALANKESLVA AGELVTTLAR ENGVQILPID
E_coli_dxr.pdb   101 IVGAAGLLPT LAAIRAGKTI LLANKESLVT CGRLFMDAVK QSKAQLLPVD

                   151        161        171        181        191
Consensus            SEHNAiFQcL pqp....... ...eqstikk iLLTaSGGPl RekpkreLqk
Conservation
Ctl2_dxr_.pdb    147 SEHNALFQCL EGR....... ...DSSTIKK LLLTASGGPL RNKSKEELQK
E_coli_dxr.pdb   151 SEHNAIFQSL PQPIQHNLGY ADLEQNGVVS ILLTGSGGPF RETPLRDLAT

                   201        211        221        231        241
Consensus            mspqqacRHP nWsMGpKIsV DSaTmmNKGL EiIEArWLFg asAsqiEalI
Conservation
Ctl2_dxr_.pdb    187 VSLQEVLRHP VWNMGPKITV DSSTLVNKGL EIIEAFWLFG LEAVEIEAVI
E_coli_dxr.pdb   201 MTPDQACRHP NWSMGRKISV DSATMMNKGL EYIEARWLFN ASASQMEVLI

                   251        261        271        281        291
Consensus            HPQSliHsMV ryqDGsiLaq mkpPsMrtPI qHtmawPeRs paigpplDFc
Conservation
Ctl2_dxr_.pdb    237 HPQSLVHGMV EFCDGTILSV MKPPSMLFPI QHVLTFPERS PAIGPGFDFL
E_coli_dxr.pdb   251 HPQSVIHSMV RYQDGSVLAQ LGEPDMRTPI AHTMAWPNRV NSGVKPLDFC

                   301        311        321        331        341
Consensus            slsaLeFaai DyDRyPslhL Akraleqkqa atcalNaANE alVaaFLAqq
Conservation
Ctl2_dxr_.pdb    287 SNRTLEFFPI DEDRFPSVHL AKRVLLEKGS MGCFFNGANE ALVHRFLAGE
E_coli_dxr.pdb   301 KLSALTFAAP DYDRYPCLKL AMEAFEQGQA ATTALNAANE ITVAAFLAQQ

                   351        361        371        381        391
Consensus            IswhqIaakl qalleqhrmq spqsleeiLS VDAeARarAq kc.....
Conservation
Ctl2_dxr_.pdb    337 ISWHQIVPKL QALVDQHRVQ SCLSLEEILS VDAEARARAQ EC.....
E_coli_dxr.pdb   351 IRFTDIAALN LSVLEKMDMR EPQCVDDVLS VDANAREVAR KEVMRLA
```
