# Supplementary figures and images for: Fosmidomycin, an inhibitor of isoprenoid synthesis, induces persistence in Chlamydia by inhibiting peptidoglycan assembly
Source: PLoS Pathog. 2019 Oct 17;15(10):e1008078. doi: 10.1371/journal.ppat.1008078 (PMC6818789; doi:10.1371/journal.ppat.1008078)

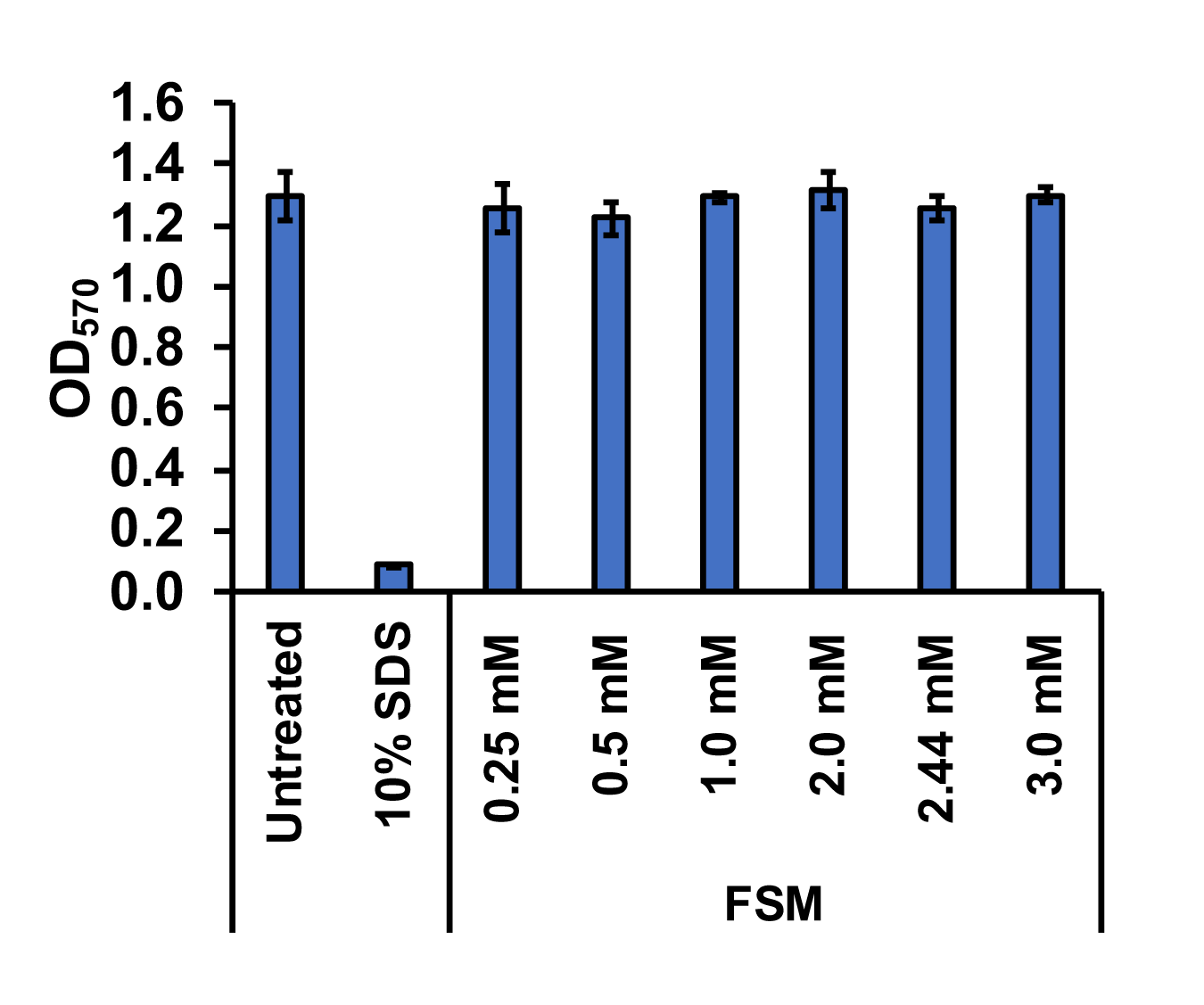

Supplement: S2 Fig — HeLa cells were incubated with increasing concentrations of FSM for 40 h and viability was assessed using the MTT assay. Data are representative of the average of triplicate samples from one experiment. 10% SDS was used as a negative control for viability. Error bars indicate +/- SD and significant (p ≤ 0.005) difference from untreated samples is indicated by an asterisk (*). (TIF) [file ppat.1008078.s002.tif]

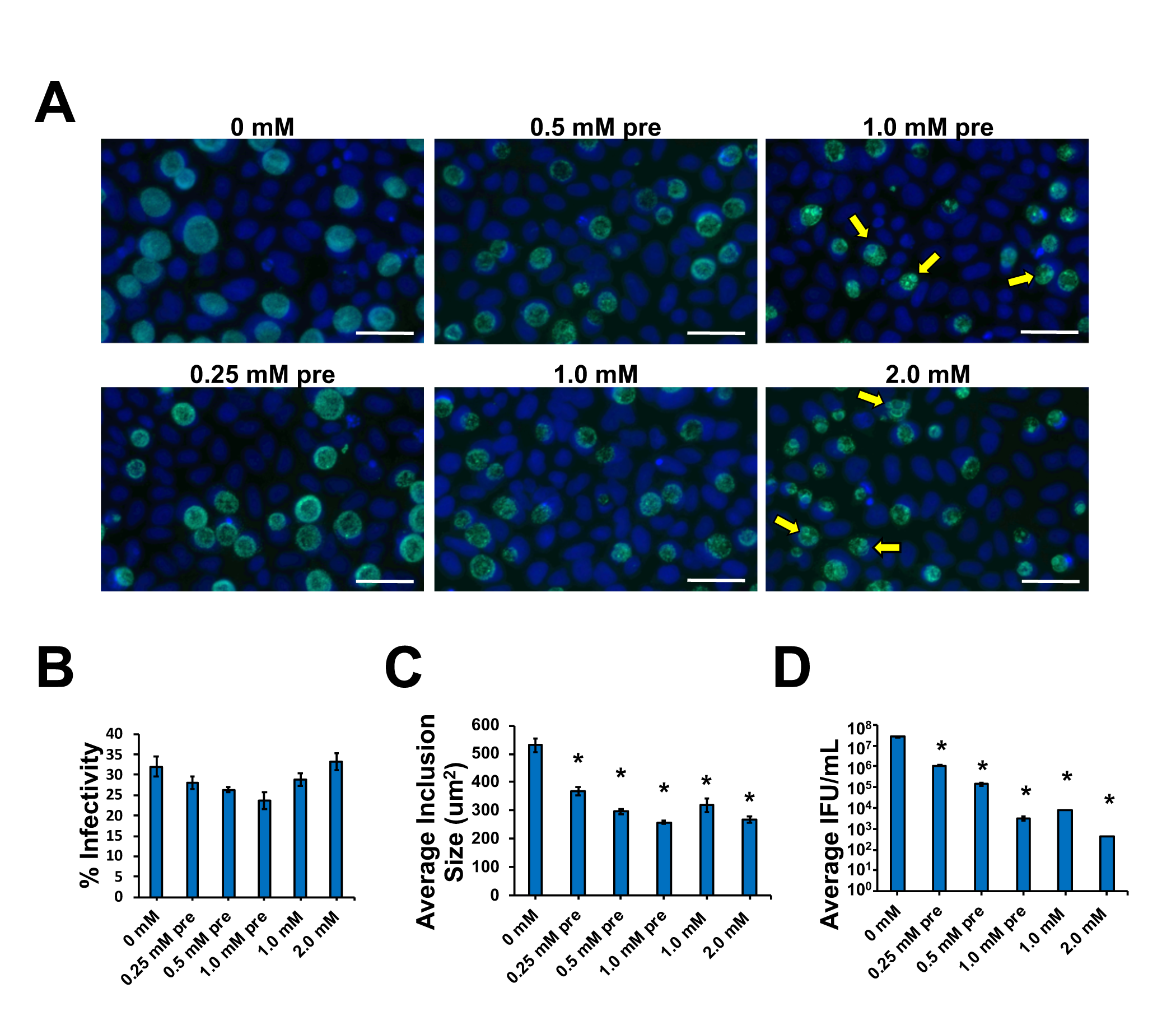

Supplement: S3 Fig — For pre-loaded samples (pre) FSM was added to tissue culture media at the time of cell plating (24 h prior to infection) and was maintained during 2 h adsorption and throughout the 40 h incubation. HeLa cells with and without FSM pre-treatment were infected with C. trachomatis at an MOI of 0.5, exposed to the indicated FSM concentrations and harvested for analysis at 40 hpi. (A) Representative 400X magnification fluorescence microscopy images of chlamydial inclusions (green) stained with BioRad anti-chlamydial LPS Pathfinder stain and HeLa cell nuclei (blue) counter-stained with DAPI. Yellow arrows indicate examples of aberrant RBs. Scale bars = 50 μm. (B) Percent infectivity was calculated by counting the number of inclusions/cell nuclei per field in 10 fields per coverslip in triplicate samples. (C) The area of 150 random inclusions per triplicate sample were measured using the spline contour tool in the Zen Blue Zeiss software package. (D) Production of infectious EBs was determined via chlamydial titer assays by subpassage. Significant (p ≤ 0.005) difference from untreated samples is indicated by an asterisk (*). Error bars indicate +/- SEM from triplicate samples and data are representative of two independent experiments. (TIF) [file ppat.1008078.s003.tif]
