# Supplementary material for: Fosmidomycin, an inhibitor of isoprenoid synthesis, induces persistence in Chlamydia by inhibiting peptidoglycan assembly
Source: PLoS Pathog. 2019 Oct 17;15(10):e1008078. doi: 10.1371/journal.ppat.1008078 (PMC6818789; doi:10.1371/journal.ppat.1008078)
Supplement: S1 Table — (DOCX) [file ppat.1008078.s004.docx]

**S1 Table. Primers used in this study.**

| **Primer Name** | **Direction** | **Sequence 5’ to 3’** | **Description** |
| --- | --- | --- | --- |
| P1 | Forward | CTCTCTGGTACCTACGAGGTTCTTTTGAAGC | P1 and P2 generate *dxr*_CT_ amplicon for cloning into pBAD24. Underlined sequence indicates KpnI and SalI restriction sites, respectively. |
| P2 | Reverse | CTCTCTGTCGACTGACATCCCACGAACA |  |
| P3 | Forward | CTCTCTGGTACCTCAACTCTGGATGTTTC | P3 and P4 generate *dxr*_EC_ amplicon for cloning into pBAD24. Underlined sequence indicates KpnI and SalI restriction sites, respectively. |
| P4 | Reverse | CTCTCTGTCGACTCTGTAGCCGGATTATC |  |
| P5 | Forward | GTAACAAAGCGGGACCAAAG | P5 and P6 primers are upstream and downstream of pBAD24 multiple cloning site. |
| P6 | Reverse | CAGTTCCCTACTCTCGC |  |
| P7 | Forward | ATCGGCTGGCGGCGTTTTGCTTTTTATTCTGTCTCAACTCTGGATGTTTCGTGTAGGCTGGAGCTGCTTCG | P7 and P8 contain upstream and downstream sequence from dxr_EC_, respectively. The underlined sequence recognizes pKD4 to amplify the kanamycin cassette. Used to generate *dxr*::*kan* linear fragment. |
| P8 | Reverse | ATTCCGGGGATCCGTCGACCCTGAAGCCCTACGCTAACAAATAGCGCGACTCTCTGTAGCCGGATTATCC |  |
| P9 | Forward | ACGATGTACAGAAACTG | P9 is upstream of *E. coli* MG1655 *dxr* and P10 recognizes the Kan cassette. Primer set used to confirm insertion of Kan cassette. |
| P10 | Reverse | GGATTCATCGACTGTGGCCG |  |
